# Risk of Mercury Exposure from Fish Consumption at Artisanal Small-Scale Gold Mining Areas in West Nusa Tenggara, Indonesia

**DOI:** 10.5696/2156-9614-9.21.190302

**Published:** 2019-03-14

**Authors:** Muhammad Junaidi, Baiq Dewi Krisnayanti, Juharfa, Christopher Anderson

**Affiliations:** 1 Department of Fish and Aquaculture, University of Mataram, Mataram, Indonesia; 2 International Research Centre for the Management of Degraded and Mining Lands, Indonesia; 3 Soil and Earth Science, Institute of Natural Resources, Massey University, Palmerston North, New Zealand

**Keywords:** ASGM, fish, mercury

## Abstract

**Background.:**

The primary environmental risk associated with artisanal small-scale gold mining (ASGM) activities in Sekotong and Taliwang is waste discharged directly into the environment. This waste contains variable concentrations of heavy metals and a high level of mercury. When these elements are released into the environment, plants and animals can be contaminated. If mercury is methylated to methylmercury, levels can increase in concentration at each level of the food chain (biomagnify). Fish are a primary risk vector for methylmercury poisoning in humans, and represent a significant source of protein for the Sekotong and Taliwang communities.

**Objectives.:**

The present study aimed to identify the concentration of mercury in fish from ASGM sites in Sekotong and Taliwang.

**Methods.:**

Descriptive research was used to describe the mercury concentrations of fish in the present study. The fish species collected for the samples represented commercially available fish most commonly consumed by the community on a daily basis.

**Results.:**

In Sekotong's ASGM area, the mercury concentration in Pilsbryoconcha exilis tissue was 596 ppb, 721 ppb for Sephia officinalis and 50% of the Euthynnus affinis samples had a high level of mercury, above the World Health Organization (WHO) maximum permissable limit for the sale of fish for human consumption of 0.5 ppb.

**Conclusions.:**

Some fish species from the studied ASGM sites had high mercury concentrations above the maximum permissible mercury concentration in edible fish tissue. The risks associated with mercury exposure from fish consumption threaten community health.

**Ethics Approval.:**

All experiments were performed in accordance with relevant local guidelines and regulations.

**Competing Interests.:**

The authors declare no competing financial interests.

## Introduction

The use of mercury (Hg) to recover gold in artisanal small-scale gold mining (ASGM) activities has contributed to widespread mercury contamination of aquatic systems in many areas worldwide.^1–5^ Across ASGM areas in Indonesia, it is common to see waste from ASGM activities discharged directly into agricultural lands and water bodies that empty into the sea.^6^

Mercury species are present in aqueous media, including methylmercury. This species can be absorbed by aquatic biota and accumulate in aquatic organisms. As a result, mercury concentrations can be magnified at higher levels of the food chain. In this system, mercury is chemically transformed, bioaccumulated and biomagnified. Thus, fish may accumulate high levels of mercury and can act as a pathway for the mercury to be consumed by people or wildlife.^2^,^7^ Concentrations of total mercury in human hair are significantly correlated with frequency of fish consumption.^8^

Human exposure to methylmercury (MeHg) occurs most frequently from consumption of marine and freshwater fish. This has become a public health challenge as fish are an important source of nutrition globally. In addition, fish are culturally important for many communities and are a profitable global commodity. The chemical form of MeHg in fish tissue has been detected in fish protein, and is not removed or destroyed by cooking or cleaning.^9^

Fish from the ASGM site in Tatelu, South Sulawesi, Indonesia have accumulated high concentrations of mercury in fish tissue due to excessive losses of mercury from mining activities to the aquatic sytem, contributing to the high biovailability of mercury in the Talawan River.^4^ Similarly, concentrations of mercury were significantly high in freshwater, estuarian and marine fishes around the area of ASGM activities in Suriname.^10^

Signs of mercury contamination have been found in seafood, molluscs, and crustaceans downstream of ASGM sites at Buru Island, Indonesia due to the geochemistry of sediments in which mercury methylation leads to highly bioavailable mercury that threatens the aquatic food chain and marine resources.^1^

High mercury concentrations from amalgamation and cyanidation tailings have been analyzed from Sekotong and Taliwang, with mean mercury concentration of the amalgamation tailings of about 3000 μg/g and greater than 1600 μg/g for the cyanidation tailings, exceeding the maximum permissible concentration for mercury in soil set by the Indonesian government of 2 μg/g.^6^ Mining in Sekotong began in mid-2009, and by 2012, 70% of Sekotong's miners had been exposed to mercury, as indicated by mean concentrations of mercury in hair samples between 7.72 – 11.61 μg/g, exceeding the recommended limit of 1 μg/g set by the World Health Organization (WHO).^11^ In 2015, Ekawanti and Krisnayanti reported that 61% of urine samples and 81% of hair samples of miners in Sekotong contained alarming levels of Hg.^12^ The participants in the study were directly exposed to mercury, either as workers (miners) or as family members (non-miners) living in the contaminated area, including children that were exposed to mercury through direct inhalation in close proximity to burning processes. The duration of exposure to mercury contaminants in Sekotong's ASGM area was an average of 5.4 years. This period was much shorter than the 14.8 years needed to show specific clinical manifestations in previous reports.^13^ Krisnayanti found similar evidence of mercury exposure around Taliwang ASGM activities where the mean concentrations of mercury in hair of exposed, indirectly exposed and non-exposed groups were 13, 1.3 and 0.56 μg/g, respectively.^14^ The ASGM sector in Taliwang has a large migrant worker population, which is an important economic support to the local community, but ASGM activities involve high mercury use and illegal mercury trading. These activities have affected the health of miners in a relatively short time, as evidenced by the high mercury residue on the bodies of miners. This indicates that mercury exposure has occurred through inhalation, however a second exposure pathway through consumption of methylmercury-contaminated food (rice) and fish could occur in the future.^6^,^15^ The primary environmental risk associated with ASGM activities in Sekotong and Taliwang is waste discharged directly into the environment. This waste contains variable concentrations of heavy metals, such as soluble complexed mercury, including mercury cyanide.

**Abbreviations**
*ASGM*: Artisanal small-scale gold mining
*WHO*: World Health Organization

Since fish are a primary risk vector of methylmercury poisoning in humans and represent a significant source of protein for ASGM communities, the consumption of fish caught around the ASGM area represents a possible human health risk. Fish is an important food for the Sekotong community as Sekotong is located along the Lombok Strait. Marine fish/seafood and freshwater fish are also an important staple food in the Taliwang diet. The aim of the present study was to identify the concentration of mercury in fish from Sekotong and Taliwang's ASGM sites.

## Methods

Fish sampling sites were located in two ASGM sites in West Nusa Tenggara Barat, in Sekotong District, West Lombok Regency at Lombok island and Taliwang District, West Sumbawa Regency at Sumbawa island. The characteristics of Sekotong and Taliwang sea waters are influenced by both the Pacific and Indian Ocean where the mass of water originating from the northern Pacific enters the Sulawesi Sea to the south of Mindanao, then enters the heart of Indonesian waters through the Makassar Strait. At the end of the Makassar Strait, this line branches into two, with water either flowing directly to the Indian Ocean through the Lombok Strait (West Nusa Tenggara) or east through the Flores Sea to the Banda Sea.^16^

The fish species collected for the samples from these areas represented commercially available fish species most commonly consumed by the community on a daily basis for the study season and the most abundant catch of the season.

ASGM activities at Sekotong began in mid-2009, and were gradually reduced in 2014, when only 15% of miners were still mining. Krisnayanti calculated that there were around 4630 of trommel (using 250–500 gram of mercury/trommel) and 150 cyanidation tanks in Sekotong, involving around 14,500 miners.^6^ In contrast, ASGM activities in Taliwang began in 2010, and mining activity has gradually increased without signs of stopping compared to Sekotong. In 2013, there were around 5000 trommel (using 250–500 gram of mercury/trommel) and 95 cyanidation tanks involving around 15,380 miners.^14^

### Sekotong sampling site

**Figure 1:**
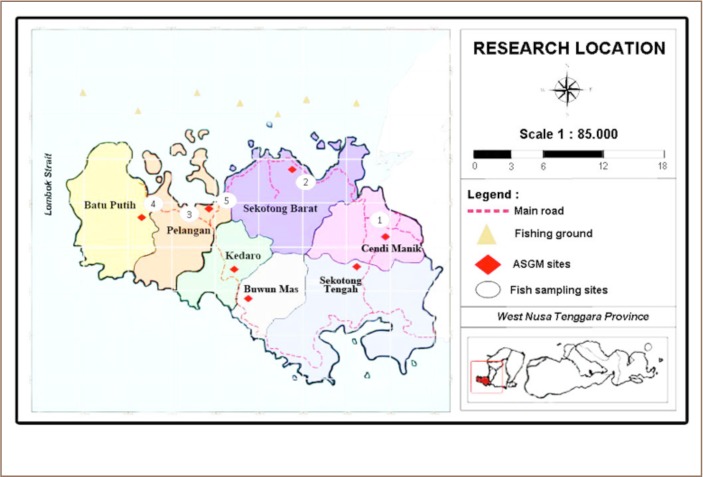
Research location: Sekotong District, West Lombok Regency, West Nusa Tenggara Province

Fish sampling at Sekotong was conducted in November 2016. There are three main traditional markets in this area, and therefore fish were sampled from all of these markets. In addition, fish were also collected from spots where fishermen most commonly sell their daily catch to local markets or local personal buyers. However, fish are also sold to markets in Mataram City and personal buyers who own restaurants in Mataram. Fish were collected from three main markets in Sekotong District: Sekotong Tengah (1), Tawun (2), and Pelangan (3) and two spots where fisherman sell their catch directly: Batu Putik (4) and Tembowong (5) (*Figure 1*). Figure 1 also shows the main ASGM spots across Sekotong District from 2009–2014. These spots were identified directly by the co-author in previous research.^17^

### Taliwang sampling site

**Figure 2:**
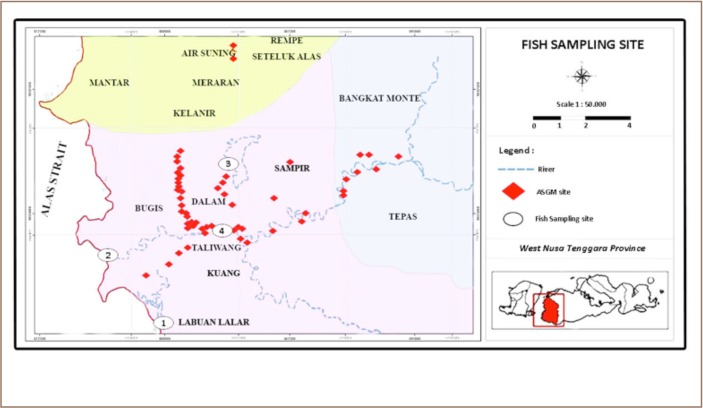
Fish sampling sites: Taliwang District, West Sumbawa Regency, West Nusa Tenggara Province

Fish sampling was conducted in Taliwang Regency in November 2016. The fish were collected from three main fishing spots: Labuan Lalar (1); Taliwang estuary (2), Lake Taliwang (3) and the main market at Taliwang (4) (*Figure 2*). Figure 2 also demonstrates ASGM activities across the Taliwang area in 2013. These spots were identified directly by the co-author in previous projects.

During sampling, fish were identified and length, size and fresh weight of fish were recorded. Mercury concentrations can increase with increased with increased fish length and age. Mercury also biomagnifies and generally increases in concentration with increasing food chain length.^11^,^18^

Each fish specimen was filleted, then dried to a constant weight at 40°C. The fish were thoroughly homogenized in a blender and bagged in plastic zip lock bags for laboratory analysis. The fish samples were analyzed at “Laboratorium Penelitian and Pengujian Terpadu” University of Gadjah Mada Yogyakarta for total mercury using a mercury analyzer (type 254) with a measuring range of 0.01–10 ppb. Mercury detection limits were 0.17 μg/g dry weight. The laboratory is accredited by the Indonesian government.

Fish samples were pre-digested overnight at room temperature with aqua regia (analytical grade nitric acid: hydrochloric acid =1:3). The next day, the samples were digested at 120°C for two hours. Samples were filtered using Whatman No. 42 filter paper into a 100 ml volumetric flask. Deionized water was used to quantitatively transfer the solution into the volumetric flask and to make up the solution to the 100 ml mark. Five (5) mL of sample solution with 5 ml of nitric acid and 1 mL of freshly prepared tin chloride (hydrochloric acid, stannous chloride) was placed into a measurement flask and prepared for measurement. For analytical precision and quality control, reagent blank solutions were analyzed in parallel with the samples. Linear calibration was prepared from 1000 mg/L of mercury standard no. 16482 as a standard reference material which was issued by TraceCERT^®^ and accredited by ISO/IEC 17025 and ISO Guide 34.^19^,^20^

A number of countries and the WHO have established a threshold for mercury concentration in fish and fish products in order to protect human health.^11^ The WHO's maximum permissible mercury concentration in edible fish tissue is 0.5 μg Hg g^−1^, which is equal to 0.5 ppm or 500 μg Hg kg^−1^, equal to 500 ppb fish tissue (wet mass basis). Mercury concentrations for the results of the present study are presented in ppb.

## Results

Some fish species from the studied ASGM sites had high mercury concentrations above the maximum permissible mercury concentration in edible fish tissue.

### Sekotong

The fishing area in West Lombok spreads across the Lombok Strait, Sekotong Bay, and the Indian Ocean. Sekotong Bay is an important fishing area especially for pearl shell, grouper fish and crayfish (lobster). In the Sekotong area, 315 marine fish specimens and mollusks were collected, predominately Loligo indica, Sephia officinalis, Siganus *sp.*, Caesio cuning, Scolopsis affinis, Valamugil seheli, Euthynnus affinis, Sardinella lemuru, Scaridae *sp.*, Acanturus *sp.*, Naso *sp.*, Lethrinus *sp.*, and Parupeneus multifasciatus, and the mollusks included Anadara granosa, Siliqua patula. No freshwater fish were collected from this area.

Previous studies have found that mercury concentrations in fish tissue were correlated with age, length, and weight of fish, but in the present study, there were no significant differences between fish length, weight and Hg concentration at a significance level of p>0.05 (one-way analysis of variance) and Table 1 indicates that there was a substantial variation in fish growth rate.^11^,^18^

**Table 1:** Characteristics of Fish Sampled at Sekotong

Tropic Category	Species	No. of specimens (n)	Weight (g)	Length (cm)
Planktivore	Anadara granosa	50	-	-
	Caesio cuning	3	340±148.49	24±4.24
	Pilsbryoconcha exilis	30	-	-
	Pomacanthus imperator	2	632±7.07	21.5±2.12
	Sardinella lemuru	35	33.75±7.41	13.38±1.11
	Siliqua patula	30	-	-
Carnivore	Chromileptes altivelis	1	27	12
	Euthynnus affinis	13	153±46.03	21.67±1.15
	Lethirinus erythracanthus	1	-	-
	Loligo indica	40	43.5±7.55	17.25±6.08
	Nemiptarus nematophorus	4	127.25±2.06	16.5±2.52
	Sephia officinalis	6	43.5±7.55	17.25±6.08
	Lethrinus erythracanthus	1	168	22
Herbivore	Ctenochaetus striatus	5	122±1.73	14±1.73
	Naso *sp.*	5	43.5±7.55	24.75±7.63
	Plectorhincus gaterinoides	2	390±1.41	23.5±0.71
	Parupeneus multifasciatus	3	162.67±1.1.5	19.67±1.1.5
	Siganus *sp.*	46	201.25±79.61	18.92±2.65
	Scaridae *sp.*	23	375±264.88	21.9±5.36
	Valamugil seheli	5	261±3.54	22.5±3.54
	Scolopsis affinis	3	246.33±110.59	22±2
Omnivore	Acanturus *sp.*	11	196.67±47.34	17.67±1.53

**Figure 3:**
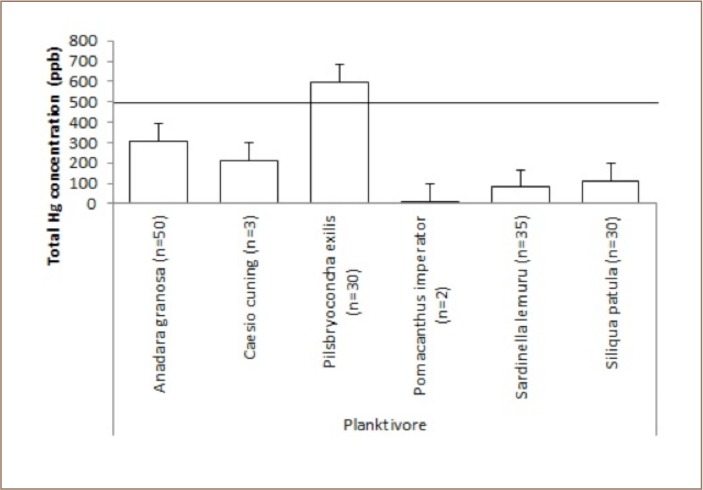
Mean levels of total mercury in planktivorous fish at Sekotong against the maximum permissible mercury concentration in edible fish tissue of 500 ppb

Among the planktivores collected in Sekotong, only Pilsbryoconcha exilis (shells) showed a high mercury concentration above the maximum permissible mercury concentration in edible fish (*Figure 3*).

**Figure 4:**
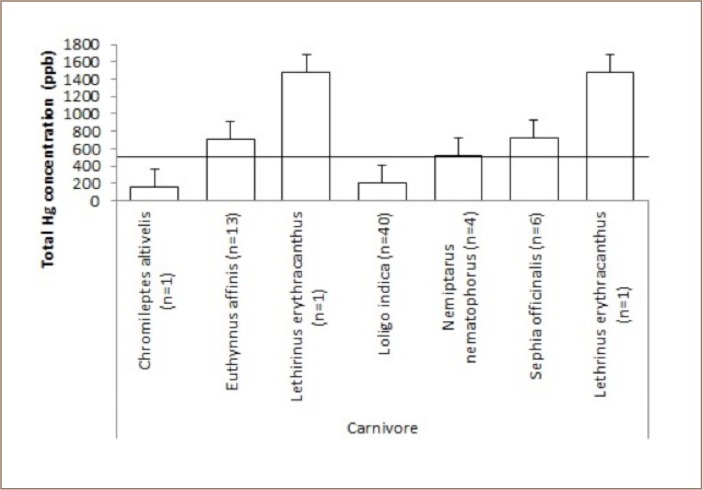
Mean levels of total mercury in carnivorous fish at Sekotong against the maximum permissible mercury concentration in edible fish tissue of 500 ppb

Of the analyzed carnivore specimens, four exhibited total Hg levels exceeding the acceptable level (*Figure 4*), and Lethirinus erythracanthus had levels 3-times higher than the maximum permissible mercury concentration in edible fish.

**Figure 5:**
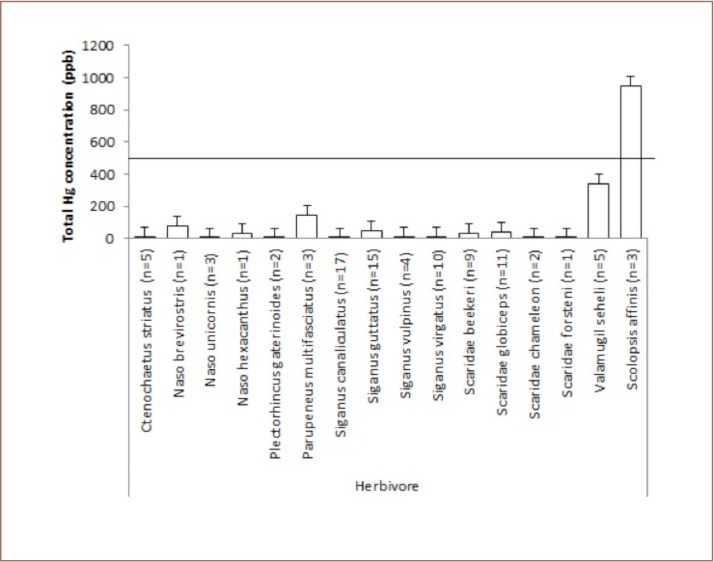
Mean levels of total mercury in herbivorous fish at Sekotong against the maximum permissible mercury concentration in edible fish tissue of 500 ppb

**Figure 6:**
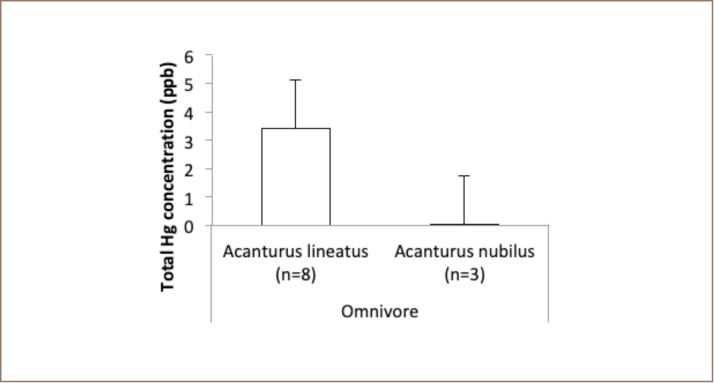
Mean levels of total mercury in omnivorous fish at Sekotong

In the herbivore category, Valamugil seheli tended to accumulate more mercury in their tissue than other species (*Figure 5*). In addition, no specimen in the omnivore category contained high mercury (*Figure 6*).

### Taliwang

In the Taliwang area, 216 marine and fresh water fish specimen were collected, including Lutjanus campechanus, Lethrinus erythracanthus, Cephalophoeis *sp.*, Siganus *sp.*, Loligo indica, Sardinella lemuru, Scaridae *sp.*, Siganus virgatus, Cheilinus trilobatus, and Valamugil seheli. Freshwater fish collected from this area included Channa striata, Oreochromis mossambikus, Anabas testudineus.

There were no significant differences between fish length, weight and mercury concentration (p>0.05, one-way analysis of variance) (*Table 2*).

**Table 2:** Characteristics of Fish Sampled at Taliwang

Tropic category	Species	No. of specimens (n)	Weight (g)	Length (cm)
Planktivore	Sardinella lemuru	22	32.40±7.09	13.1±1.14
Herbivore	Cephalophoeis mimiata	3	256±0.00	21.5±0.50
	Channa striata^*^	2	395.50±7.77	30.75±0.35
	Cheilinus trilobatus	3	43.66±0.57	23.16±0.28
	Epinephelus *sp.*	8	233.33±74.93	20.07±2.61
	Lutjanus *sp.*	5	375.66±71.06	24.00±1.00
	Lethrinus erythracanthus	3	396.66±32.14	22.00±2.00
	Loligo indica	30	43.5±7.55	17.25±6.08
	Terapon jarbua	4	25.00±1.42	8.70±0.53
Carnivore	Chanos chanos	3	20.66±2.08	10.52±0.85
	Rasbora hengeli	9	12.02±0.36	7.97±0.28
	Siganus *sp.*	11	180.66±79.95	18.16±3.51
	Scaridae globiceps	6	330.33±95.11	21.00±2.00
	Valamugil seheli	7	22.62±7.59	10.2±1.56
Omnivore	Anabas testudineus^*^	10	61.20±4.92	11.70±0.97
	Geres punctatus	36	15.98±0.27	7.96±0.17
	Leiognathus equulus	25	10.75±0.46	6.58±0.46
	Oreochromis mossambikus^*^	11	118.67±63.85	15.5±2.78
	Silago sihama	2	52.50±3.53	16.60±0.85

^*^freshwater fish

**Figure 7:**
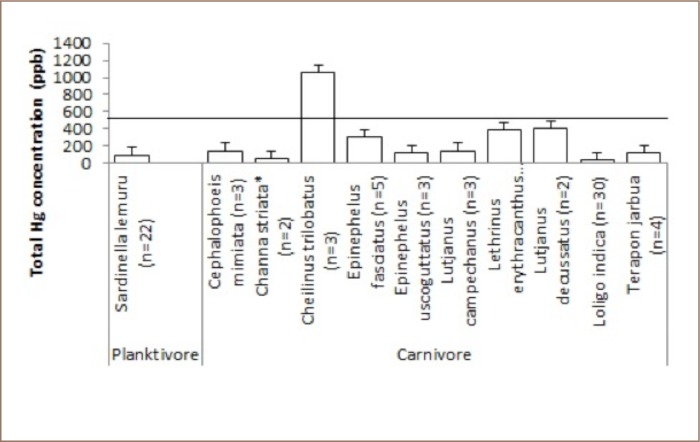
Mean levels of total mercury in fish from the planktivore and carnivore categories at Taliwang against the maximum permissible mercury concentration in edible fish tissue of 500 ppb

Among the carnivorous fish collected in Taliwang, only Cheilinus trilobatus showed a high concentration of mercury, twice the maximum permissible mercury concentration for edible fish (*Figure 7*).

**Figure 8:**
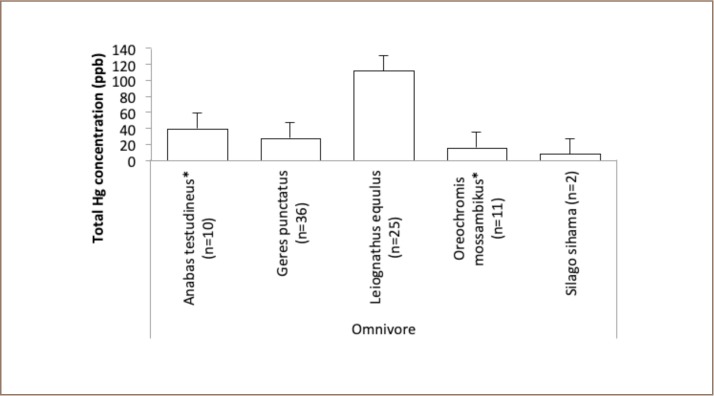
Mean levels of total mercury in omnivorous fish at Taliwang

**Figure 9:**
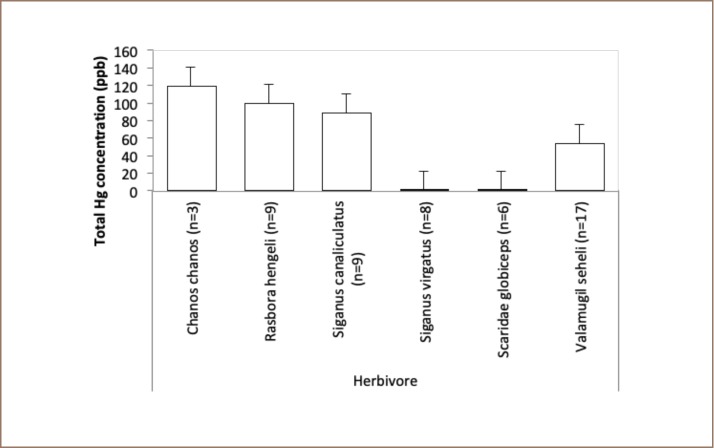
Mean levels of total mercury in herbivorous fish at Taliwang

No samples in the omnivore category of marine or freshwater fish *(Figure 8*) or herbivore (*Figure 9*) category contained high concentrations of mercury.

## Discussion

Fish and seafood are important dietary constituents because they are a high quality source of protein, vitamins, minerals and omega-3 fatty acids. However, fish have become a major source of methylmercury intake in humans. The main concern is that mercury content in fish can be biomagnified rapidly depending on the environmental parameters in water and sediment. Although the mercury concentration in water bodies in these areas was not measured in the present study, ASGM activities have been recognized as a key factor contributing mercury emmisions to the aquatic system. In general, more than 90% of the mercury in fish occurs as methylmercury, but methylmercury contents can vary considerably between species. In aquatic systems, mercury is transformed into methylmercury by microorganisms and abiotic reactions. Methylmercury becomes increasingly concentrated in the marine food chain, in a process referred to as biomagnification, and can reach extremely high levels in predatory fish such as swordfish, tuna, king mackerel and shark. As a result, the risk of human exposure to methylmercury through consumption of large predator fish may be elevated. In the sampling sites in the present study, however, large predator fish are not often part of the catch of traditional fisherman. The small size of fish collected in the present study represents the fish most commonly consumed in this area.

Around 10% of the fish samples from Sekotong contained very high methylmercury concentrations. These results indicate that bioaccumulation of mercury had occurred in fish in Sekotong, particularly for fish in the planktivore and carnivore categories. In the present study, the mercury concentration in Pilsbryoconcha exilis (shell, locally kijing (596 ppb) (*Figure 3*) was above the permissable limits for human consumption which is 0.5 μg Hg g.^11^ The habitat for this organism is mud/sediment that may possibly contain mercury from mine waste discharged to rivers, while biotic demethylation reactions usually occur in sediment. This indicates that mercury contamination may have reached the estuary where this organism is found. Attention should be paid to the consumption of Euthynnus affinis (small tuna, locally tongkol), as 50% of the samples in the present study had high levels of methylmercury in their tissue. In addition, Sephia officinalis (squid, locally cumi) had a methylmercury concentration of 721 ppb (*Figure 4*). Even though Euthynnus affinis is not a demersal fish and occurs in open waters, it remains close to the shoreline, and its young may enter bays and harbors for feeding. As shown in Table 1, even though there was no statistically significant relationship between mercury concentration, size and length of fish, there was a tendency for higher mercury concentrations in fish tissue in bigger fish (around 20 cm), such as Scolopis affinis (24 cm), Euthynnus affinis (21.67 cm), and Lethrinus erythracanthus (18 cm). Most of the fish samples were small.

Results of the Taliwang samples suggest that marine and freshwater fish in this area are below the permissable limits for consumption by the community. In the Taliwang samples, only one marine fish from the carnivore category had a high mercury concentration, Cheilinus trilobatus (1063 ppb) (*Figure 7*). Attention should be paid to the consumption of this species, as it may pose a health risk. This fish is in the predator category and occasionally feeds in seagrass beds, mainly on benthic invertebrates such as mollusks and crustaceans. Unfortunately, no mollusks and crustaceans were sampled from this area to support this finding. Taliwang residents are concerned about contaminated freshwater fish from Lake Taliwang, due to the many gold mining and processing activities around Lake Taliwang. However, the results showed no evidence of mercury contamination in freshwater fish from Lake Taliwang, even for large fish such as Channa striata (*Figure 7*) or Oreochromis mossambikus and Anabas testudineus (*Figure 8*) (30.75, 15.5 and 11.70 cm), respectively. These findings could be due to differences in fish habitat use within the lake and growth rates as a function of lake size.^21^ These three species are the most commonly consumed species in Taliwang.

Consumption of fish provides excellent nutrition, but fish may contain levels of mercury above the WHO permissible limits for human consumption.^11^ Sensitive groups in the population can best protect their health by avoiding fish species that may contain high levels of mercury. Vulnerable groups like pregnant women, breast feeding mothers and children up 3 years of age should be advised not to consume potentially contaminated fish. Frequent consumption of seafood can increase the risk of mercury exposure, which is potentially dangerous to vulnerable groups. Even though rates of fish consumption were not measured in the present study, the authors observed that the average daily fish intake in these areas was high, as fish are a main dietary component in the communities of Sekotong and Taliwang. It was suggested that the most at risk group are women of childbearing age and children who should keep mercury intake below 0.2 ug/kg body weight per day. They should eat locally caught fish only every second day. It is a limitation of this study that no fish were sampled from control sites, therefore it is not possible to definitively say that the elevated mercury levels were due to ASGM activity in the area, although it is probable. Miners and the surrounding community have become aware of the risks associated with mercury exposure and mining activities, but programs are needed to increase awareness and to address community concern over the risks associated with mercury exposure and fish consumption.

## Conclusions

A few fish species from the sites in the present study had mercury concentrations above the maximum WHO permissible mercury concentrations in edible fish tissue. The risks associated with mercury exposure and fish consumption threaten the health of residents of these communities.
